# *In-vivo* analysis of visible light cure calcium hydroxide, mineral trioxide aggregate and platelet-rich fibrin with and without laser therapy for direct pulp capping

**DOI:** 10.6026/9732063002001111

**Published:** 2024-09-30

**Authors:** Aditee Agrawal, Rana K. Varghese, Naveen Kumar Gupta, Nishant Choubey, Astha Dubey, Swati Priya

**Affiliations:** 1 Department of Conservative Dentistry & Endodontics, New Horizon Dental College and Research Institute, Sakri, Bilaspur, Chhattisgarh - 495001, India; 2 Department of Oral and Maxillofacial Prosthodontics and Implantology, New Horizon Dental College and Research Institute, Sakri, Bilaspur, Chhattisgarh - 495001, India

**Keywords:** Laser, Direct Pulp Capping, Mineral Trioxide Aggregate, Platelet Rich Fibrin, Calcium Hydroxide

## Abstract

The clinical and radiographic outcomes of direct pulp capping using visible light cure calcium hydroxide, Mineral Trioxide Aggregate, and Platelet-Rich Fibrin, with and without pre-treatment with a low-level diode laser is of interest to dentist. One hundred and twenty patients (18-48 years) with accidental minimal pulp exposures less than 1 mm2 were randomized into two primary groups: laser pre-treatment and no laser pre-treatment, each further divided into three sub-groups based on the capping material. Clinical and radiographic assessments were conducted at 7^th^ day, 1^st^, 6^th^ and 12^th^ months. The combination of low-level diode laser and mineral trioxide aggregate with platelet-rich fibrin demonstrated superior clinical outcomes compared to other groups. Radiographic analysis showed significant differences in dentin bridge thickness among groups. While all tested materials exhibited promising results, further research is necessary to optimize treatment protocols and ensure long-term clinical success with a specific focus on dentin bridge formation and the potential influence of laser pre-treatment.

## Background:

The preservation of pulp vitality is a fundamental objective in dentistry, as healthy pulp tissue possesses inherent regenerative capabilities that allow for healing even after carious exposure, provided the inflammation is reversible [1]. Maintaining vital pulp is crucial as it offers superior protection against masticatory forces compared to endodontically treated teeth, which exhibit lower survival rates, especially in molars [1, 2]. The first documented pulp capping procedure, performed in 1756 by Phillip Pfaff, involved placing a small piece of gold over an exposed vital pulp to stimulate healing [3]. Historically, the success rates of pulp capping have been low, with one retrospective study reporting a 37% success rate at 5 years, decreasing to 13% at 10 years, and another study showing success rates of only 32% and 35% for direct pulp capping and partial pulpotomy, respectively, after one year [4, 5]. Due to these unpredictable outcomes, Vital Pulp Therapy (VPT) was traditionally recommended only for immature teeth and those with accidental traumatic pulp exposures [5]. However, recent studies and systematic reviews have reported improved outcomes for VPT when performed with bioceramic materials [6]. Pulp exposure can result from dental caries, mechanical injury, or traumatic events. According to the American Association of Endodontics (2003), direct pulp capping (DPC) is defined as the treatment of mechanical or traumatic pulp exposure by sealing the wound with a biomaterial placed directly on the exposed pulp to facilitate reparative dentin formation and maintain pulp vitality [7]. The European Society of Endodontology has emphasized the need for research in pulpitis control and conservative pulp management to reduce root canal treatments and promote biomimetic approaches [8]. Various materials have been used for direct pulp capping, with calcium hydroxide being a consistent choice due to its clinical success [9]. A novel light-cured calcium hydroxide formulation has recently been introduced, offering favorable viscosity and precise application, with controllable setting time through light activation [9]. Mineral Trioxide Aggregate (MTA), bioactive, biocompatible calcium silicate cement, has also gained prominence due to its ability to stimulate human dental pulp cell proliferation, differentiation, and rapid dentin bridge formation [10]. Additionally, platelet-rich fibrin (PRF), introduced by Choukroun in 2001, offers a three-dimensional fibrin network enriched with components that facilitate soft and hard tissue regeneration [11]. Laser therapy is emerging as a promising non-pharmacological approach for DPC, with studies indicating its antibacterial, biostimulatory, hemostatic, and wound-healing properties. For instance, a clinical study by Yazdanfar *et al.* reported a 100% success rate with diode low-level laser therapy in DPC after one year, compared to a 60% success rate in the control group [12]. This study aims to compare the efficacy of various direct pulp capping materials-light-cured calcium hydroxide, mineral trioxide aggregate and platelet-rich fibrin-in stimulating dentin bridge formation, evaluated through radiographic assessment and clinical pulp testing during follow-up appointments.

## Materials and Methods:

This research was conducted in the Department of Conservative Dentistry and Endodontics at New Horizon Dental College and Research Institute, Sakri, Bilaspur, Chhattisgarh, India. The study received approval from the Institutional Ethical Committee and informed consent was obtained from all participants. The study included 120 teeth with accidental pulp exposures in patients aged 18 to 48 years, selected from the department's outpatient clinic and dental camps organized by the Department of Public Health Dentistry.

## Inclusion criteria were:

Pulp exposure less than 1 mm^2^ within 24 hours, systematically healthy and cooperative patients, permanent teeth with deep carious lesions, vital pulp with positive response to thermal and electric pulp tests, sensitivity to cold food or food lodgement and no radiographic evidence of periapical pathology. Exclusion criteria included: symptomatic teeth, inability to isolate with a rubber dam, teeth with irreversible pulpitis, large exposures, uncontrolled bleeding, non-restorable teeth and allergies to capping materials, pathologic mobility, radiolucency in periapical or furcation areas, external or internal resorption and canal obliteration.

Following a comprehensive patient history and pulp vitality tests, teeth were cleaned and prepared. Cavity preparation involved caries excavation using a sterile round bur (Mani, India) under water coolant, with the deepest carious tissue adjacent to the pulp removed last under magnification loupes (x3.5). Rubber dam isolation was used to minimize bacterial contamination. The cavity was disinfected with 3% sodium hypochlorite and hemostasis was achieved with a dry cotton pellet.

## For Group I (laser group):

[1] Group I (a): The exposed pulp was treated with a low-level diode laser, followed by a 2 mm layer of light-cured calcium hydroxide, a 2 mm layer of chemically-cured glass-ionomer cement (Fuji II GC, Tokyo, Japan) and a composite resin restoration (Tetric N-Ceram, IvoclarVivadent, Liechtenstein).

[2] Group I (b): A 2 mm layer of MTA, covered with a moist cotton pellet, and a temporary restoration (Orafil-G) was placed. After 24 hours, the restoration was completed as in Group Ia.

[3] Group I (c): PRF, prepared according to Choukroun's technique, was applied to the pulpal wound, followed by a 2 mm layer of MTA, and restored as in Group Ib.

For Group II (non-laser group), the procedure was identical to Group I but without laser irradiation.

Radiographs were taken to evaluate reparative dentin formation. Clinical success was defined by normal pulp sensitivity, absence of pain, tenderness, swelling, sinus tracts, pathological mobility, and, in anterior teeth, no discoloration. Follow-up was conducted at 7 days, 1 month, 6 months and 12 months. Statistical analysis was performed using Mann-Whitney, Kruskal-Wallis, Friedman, and Chi-square tests.

## Results:

**Figure 1:**
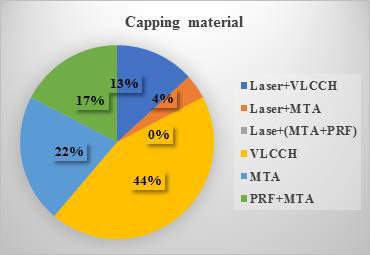
Total failure outcomes in terms of capping material

Data were collected through clinical examinations (cold, heat, and electric pulp tests) and radiographic assessment of dentin bridge thickness at 7-day, 1-month, 6-month, and 1-year follow-ups (100% patient recall). Data were entered into Microsoft Excel 365 from Word for Office 365 and analyzed using SPSS Statistics 23.0 (IBM Corporation, Armonk, NY, USA).To compare the six groups, Kruskal-Wallis and Friedman tests were employed. A statistically significant difference (p < 0.001) was observed among the treatment groups. The chi-square test was used to assess radiographic outcomes, with a p-value of <0.001 indicating significant differences in dentin bridge thickness among groups over time. The overall success rate of direct pulp capping was 80.83% (97/120 cases). Table 1 presents the success rates of the two primary groups after one year. Table 2 details the success rates of individual groups after one year. Figure 1 illustrates total failure outcomes by capping material. Table 3 compares dentin bridge thickness (DBT) among treatment groups. The laser group demonstrated statistically significantly higher success rates than the non-laser group based on mean rank analysis. Statistical analysis of clinical and radiographic variables revealed significant differences among groups.

## Discussion:

Advances in understanding the healing and regenerative capacity of irreversibly inflamed pulp have stimulated research into minimally invasive treatments for mildly inflamed pulp [22]. Inclusion criteria for this *In vivo* study were designed to mimic routine clinical practice. Given the conflicting literature regarding the impact of age on pulp-capped tooth success, with some studies reporting higher healing potential in younger patients and others finding no correlation with age [25], this study included eligible patients aged 18-48 years. Teeth with proximal caries were also included provided optimal isolation and coronal sealing were achieved [14]. While limited data exists on the success of direct pulp capping (DPC) for proximally exposed caries, based on prior research [12] and considering the minimal pulpal exposure of less than 1 mm^2^, a one-year clinical follow-up was deemed sufficient for provisional prognosis of final restorations. This timeframe also allowed for the evaluation of DPC efficacy using different materials. A standardized protocol, encompassing clinical and radiographic parameters (CT, HT, EPT and DBT)was established prior to follow-up analysis.

A variety of conventional and newer materials used for DPC have been recommended for use in DPT [15, 16, 17, 18, 19, 20, 21, 22 and 23]. Calcium hydroxide (CH) is the gold standard for pulp capping due to its beneficial properties. To address CH's limitations, light-cured calcium hydroxide (LCH) was introduced, offering improved handling, strength, and durability. LCH exhibits comparable antimicrobial efficacy to CH and demonstrates superior physical properties. Additionally, LCH materials have shown high bioactivity due to their porous structure, facilitating ion release [1-10, 12, 17]. PRF, an autologous material, is biocompatible and increasingly used in pulp capping due to favorable outcomes. Combined with MTA, it enhances pulp capping success. PRF's platelet concentrate promotes pulp cell regeneration and dentin genesis, acting as a bio-scaffold for revascularization and growth factor reservoir [24, 25]. Recently, MTA has become an alternative material for direct pulp capping. A previous clinical trial by Marques et al reported that MTA and Endocem, which is an MTA-derived Portland cement, showed 95.5% and 90.5% success rates, respectively [18, 26,[Bibr R27]].

## Conclusion:

This study provides valuable insights into the potential of different materials and approaches for direct pulp capping. While all three materials investigated demonstrated promising success rates, further research is required to optimize treatments and ensure long-term success for patients. By incorporating various recent advancements, future research in DPC can improve treatment efficacy, provide more predictable outcomes, and offer personalized solutions for preserving pulp vitality in teeth with minimal exposures like considering advancements in materials, techniques, and assessment methods.

## Limitations of the study:

Limitations of this study include a single LLLT application, small sample size, and reliance on less definitive diagnostic methods such as traditional pulp vitality tests. Pulse oximetry, a more reliable vitality assessment tool, could have enhanced the study's outcomes. Future research should explore intermittent LLLT, larger sample sizes, and advanced imaging techniques like CBCT. Additionally, longer follow-up periods and standardized regenerative DPC protocols are essential for drawing definitive conclusions.

## Figures and Tables

**Table 1:** Presents the success rates of the two primary groups after one year

**Group**	**Group - I: with laser**	**Group - II: without laser**
Cases:	(56/60)	(41/100)
Success rate:	93.33%	68.33%

**Table 2:** Details the success rates of individual groups after one year

	Group - I(a) LLDL + VLCCH	Group - I(b) LLDL + MTA	**Group - I(c) LLDL + (MTA+PRF)**	**Group - II(a) VLCCH**	**Group - II(b) MTA**	**Group - II(c) MTA +PRF**
Cases:	(17/20)	(19/20)	(20/20)	(10/20)	(15/20)	(16/20)
Success rate:	85%	95%	100%	50%	75%	80%

**Table 3:** Compares dentin bridge thickness (DBT) among treatment groups.

	**No hard tissue formation**	**Partial hard tissue formation**	**Complete hard tissue formation**
Group - I(a)	15%	55%	30%
Group - I(b)	5%	10%	85%
Group - I(c)	0%	10%	90%
Group - II(a)	50%	40%	10%
Group - II(b)	30%	35%	35%
Group - II(c)	25%	10%	65%
